# Fluorescence lifetime distribution in phakic and pseudophakic healthy eyes

**DOI:** 10.1371/journal.pone.0279158

**Published:** 2023-01-06

**Authors:** Chantal Dysli, Muriel Dysli, Sebastian Wolf, Martin Zinkernagel

**Affiliations:** Department of Ophthalmology, Inselspital, Bern University Hospital and Department of BioMedical Research, University of Bern, Bern, Switzerland; Wrocław University of Science and Technology, POLAND

## Abstract

**Purpose:**

To investigate the influence of the lens status and to describe fundus autofluorescence lifetimes (FLT) in a large cohort of healthy eyes across a wide age range.

**Materials and methods:**

FLT data were acquired from healthy phakic and pseudophakic eyes using fluorescence lifetime imaging ophthalmoscopy (FLIO). Retinal autofluorescence was excited with a 473 nm laser and emitted autofluorescence was detected in a short and a long spectral channel (SSC: 498–560 nm; LSC: 560–720 nm).

**Results:**

141 healthy eyes from 141 participants (56 ± 18 years) were included. The shortest mean FLTs were measured within the macular center, followed by the temporal inner and outer ETDRS (Early Treatment Diabetic Retinopathy Study) grid segments, and the remaining areas of the inner and the outer ETDRS ring. In phakic participants (81%), mean, short and long FLTs correlated with the age (SSC: r^2^ = 0.54; LSC: r^2^ = 0.7; both p<0.0001) with an increase of about 33 ps in the SSC resp. 28 ps in the LSC per decade. In pseudophakic subjects (19%), mean FLTs only correlated with age in the long spectral channel (r^2^ = 0.44; p = 0.0002) but not in the short spectral channel (r^2^ = 0.066; p = 0.2).

**Conclusions:**

Fundus autofluorescence lifetimes are age dependent. FLTs in the SSC are more susceptible to lens opacities but less dependent on age changes, whereas FLTs in the LSC are largely independent of the lens status but display a higher degree of age dependency.

**Study registry:**

ClinicalTrials.gov NCT01981148.

## Introduction

Fluorescence lifetime imaging ophthalmoscopy (FLIO) measures the decay- or lifetime (FLT) of fluorophores of the retina upon excitation and therefore provides differentiated information about the composition and distribution of endogenous retinal fluorophores, and about their molecular environment [1].

Briefly, a blue laser light is used to excite various retinal fluorophores such as lipofuscin, melanin or endogenous carotenoids. Thereby, these molecules gain a higher level of energy, and—after a specific timespan—release their energy partly in form of light with longer wavelengths in order to reach the ground state again. The technique is generally known as fundus autofluorescence intensity measurements (FAF), and has been used in ophthalmology either qualitatively or quantitatively. Within the retina, one of the main fluorophores detected by FAF is lipofuscin [2–5]. Instead of measuring autofluorescence intensity, FLIO investigates the time between excitation and emission of endogenous fluorophores which is specific for each molecule and its environment [6,7]. Using FLIO, weak fluorophores can be detected if they feature individual lifetimes [8,9]. One example of a weak fluorophore is thought to be the macular pigment. It is composed of lutein and zeaxanthin two carotenoid pigments of the xanthophyll subclass, which present in high concentrations in the retina, especially in the macula [4,10,11]. Macular pigment appears hypoautofluorescent in FAF intensity measurements due to absence of autofluorescence properties using 488 nm excitation [12,13]. In FLIO however, macular pigment provides good contrast with very short lifetimes [12].

In the last years, the FLTs of various retinal diseases have been characterized [6,7,12,14,15]. FLIO has been shown to provide additional information to other imaging modalities such as characterization of retinal deposits over time in Stargardt disease [16], or the area of remaining photoreceptors in choroideremia or retinitis pigmentosa [17,18].

However, by now the influence age in relationship with the crystalline lens on the measured FLT is not clear and incompletely understood. Therefore, this study aims to investigate the influence of the intraocular lens on FLT by comparing FLIO in phakic and pseudophakic healthy eyes over a broad age range. This study provides an in-depth characterization of FLTs and individual FLT parameters in a large cohort of healthy subjects of various age groups.

## Materials and methods

### Subjects and procedures

FLT data of healthy eyes of subjects recruited at the outpatient department of ophthalmology at the University Hospital in Bern, Switzerland were analyzed. Informed consent was obtained from all participants before study entry after explanation of the nature and possible consequences of the study. The study was performed according to the tenets of the Declaration of Helsinki and is approved by the institutional review board (Kantonale Ethikkommission für die Forschung, Murtenstrasse 31, 3010 Bern, Switzerland) and registered at ClinicalTrials.gov (NCT01981148).

All participants underwent a clinical ophthalmic routine examination which included assessment of best corrected visual acuity (BCVA, Early Treatment Diabetic Retinopathy Study (ETDRS) letters) [19], intraocular pressure by air tonometry, maximal pupil dilation with tropicamid 0.5% and phenylephrine hydrochloride 2.5% followed by a general ophthalmic examination.

Only one eye per subject was included in the study. Eyes with pathological findings (anterior and/or posterior eye segment) or with refractive errors over ± 4 diopters were excluded from the study. Subjects with clinically significant cataract were not included in this study.

### Fluorescence lifetime imaging ophthalmoscope

FLIO measurements were performed using a fluorescence lifetime imaging ophthalmoscope based on a Heidelberg retina angiograph (HRA) Spectralis system (Heidelberg Engineering, Germany). Detailed description of the FLIO technique and the corresponding laser safety calculations have been published in detail in previous reports [6,16,20].

In short, the fundus of the eye was scanned with a focused pulsed laser beam of 473 nm wavelength and 80 MHz repetition rate. The optical pulse width was about 60 ps full width at half maximum. The average radiation power in the eye was maximally 90 μW which lays below the safety limits calculated by ANSI and IEC. The emitted fluorescence is fed back through the scanner, and detected through a confocal pinhole. It was further split in a short-wavelength (498 to 560 nm, SSC) and in a long-wavelength (560 to 720 nm, LSC) spectral channel. The signals are detected by single-photon hybrid detectors, and the resulting electrical pulses recorded by two parallel time correlated single photon counting modules (TCSPC, Becker&Hickl, Berlin, Germany) [21]. Synchronization with the scan is performed by frame-clock and line-clock pulses from the scanner. To track and correct for eye movements in real time and to ensure that every single fluorescence photon is registered at the correct spatial location, high-contrast confocal infrared reflection images were recorded simultaneously with the fluorescence data. The images from the infrared channel are used to track eye motion and provide an x-y correction vector for the TCSPC registration. The resulting data are arrays of 256 x 256 pixels, each containing photon numbers in 1024 time-channels for consecutive times in the fluorescence decay.

### Imaging procedure

FLIO images were acquired in a dark room to avoid background light entering the detectors. All examinations were performed with maximally dilated pupils. Participants were asked to look at an internal fixation target to maintain stable vision and avoid eye movements. To ensure an adequate fitting procedure, a minimal amount of 1’000 photons per pixel was recorded within the macular center which resulted in an acquisition time of approximately two minutes. The standard FLIO image covers a central field of 30° (about 8,6 mm x 8,6 mm) of the ocular fundus and consists of 256 x 256 pixels, resulting in a spatial resolution of 34 μm. In parallel, a fundus autofluorescence intensity image was obtained.

### Fluorescence lifetime data analysis

The acquired data were analyzed with the software SPCImage 4.6 (Becker+Hickl, Berlin, Germany) [21]. SPCImage fits the decay data in the individual pixels by a weighted least-square procedure. A shift parameter accounts for possible changes of the optical path length, and an ’incomplete decay’ option compensates for fluorescence remaining from previous excitation pulses. The instrument-response function (IRF) accounts for the internal path of the light beam of the device. It is measured once, deposited and recalled for data analysis. The fitting algorithm is performed automatically by the software. It convolutes the model function with the IRF, determines the differences between the photon numbers in the TCSPC time channels and the data points of the convoluted model function, and calculates a weighted error sum of the squared differences. The weight function is 1/(n(t)-f(t))^2^, with n = photon number, f(t) = model function. The weighting account for the fact that the photon numbers in the time channels are Poisson-distributed [1]. The decay parameters (i.e. the lifetimes and amplitudes of the decay components) are then optimized by a Levenberg-Marqard algorithm until the sum of the weighted differences is at minimum. The quality of the fit is shown by the usual chi^2^ parameter, which is available for each individual pixel.

As the number of detected photons per pixel *in vivo* is limited, SPCimage offers spatial binning of FLIO data to increase the accuracy of the fitting procedure. The binning function uses the fact that the point-spread function in image data is normally spatially oversampled. That means that the decay data in pixels within the area of the point spread function are highly correlated. The binning function makes use of this by overlapping binning of the decay data within an area around the current pixel position. Thus, the number of pixels remains unchanged, but the number of photons for the analysis of each pixel is increased. For analysis of our FLIO data we used binning within a 3 by 3 pixel area or one pixel around the current one (binning factor 1 in SPCImage) for every single pixel within the 256x256 frame. This results in an increase of the photon number by a factor of 9 per pixel. However, the total number of pixel does not change as the binning procedure represents a moving average over the whole image.

We used a double-exponential model to analyze our data. Thereby, the primary outcome measurements were a short and a long decay times (*T1* and *T2*) and their respective intensities (amplitudes) *α1* and *α2*. The sum of the two amplitudes is 100%. An amplitude weighted mean fluorescence lifetime (*Tm*) was evaluated according to following equation:

$$Tm=\frac{\alpha 1*T1+\alpha 2*T2}{\alpha 1+\alpha 2}$$ (1)

Most likely, the decay data and the investigated tissue contain more than these two components [21]. However, the number of photons per pixel or per binning area is limited in *in vivo* FLIO data as the total acquisition time per image and eye is limited to one to two minutes. This results in a much lower photon count than in cuvette-based fluorescence-lifetime measurements. Based on available data, the double-exponential model seems to be appropriate as models with higher numbers of components are not suitable for the present data. Moreover, FLIO is mainly focusing on the amplitude-weighted mean lifetime, *Tm*, as shown above. More complex models may give hints to additional decay components but do not deliver differences in *Tm*. The way the data is fitted represents a model to incorporate the parameter with the most influence to the data analyzed (99.5–99.6% of the data are representatively included in the data analysis). However, the data fit using discrete decay parameters does not exactly represent the continuous sum of decay parameters.

For quantitative analysis of measured fluorescence lifetimes, the data from SPCImage were imported into the ‘FLIO reader’ software (ARTORG Center for Biomedical Engineering Research, University of Bern, Bern, Switzerland). This software allows autofluorescence intensity and color-coded fluorescence lifetime images to be displayed in an adjustable intensity overlay. To compare different areas of the retina, a standard ETDRS grid was used with circle diameters of 1 mm for the central area (C), 3 mm for the inner ring (IR), and 6 mm for the outer ring (OR). For analysis of FLTs of the optic nerve head, a circle with 1 mm diameter was used. Mean values for all FLT parameters were built for every grid area and the optic nerve head.

### Statistical analysis

Data were processed using Excel (Microsoft, Redmond, WA, USA). Prism GraphPad commercial software package (Prism 6; GraphPad Software, Inc., La Jolla, CA, USA) was used to perform statistical analysis. If data were normally distributed due to D’Agostino and Pearson omnibus normality test, data were compared using a two-tailed T-test with a confidence interval of 95%. In non-parametric data, Wilcoxon matched pairs test was applied. In linear regression models, the r^2^ value indicates the coefficient of determination and represents the goodness of fit. Results with P values below 0.05 were considered to be statistically significant.

## Results

Fluorescence lifetime data of 141 eyes from 141 subjects (44.6% female) were analyzed. The age ranged from 21 to 91 years with a mean of 56 years. 81% of the eyes were phakic. Further details can be found in Table 1 and in S1 Fig.

**Table 1:** Descriptive data of the included subjects.

	total	phakic	pseudophakic
**Number**	141	114 (81%)	27 (19%)
**age mean +/- SD (years)**	56 ± 18	52 ± 17	73 ± 11
**age median (years)**	59	55	75
**range**	21–91	21–81	49–91
**sex (f/m)**	63 f / 78 m	51 f / 63 m	12 f / 15 m

female (f), male (m), standard deviation (SD).

### Fluorescence lifetimes distribution within the ocular fundus

**Fig 1:**
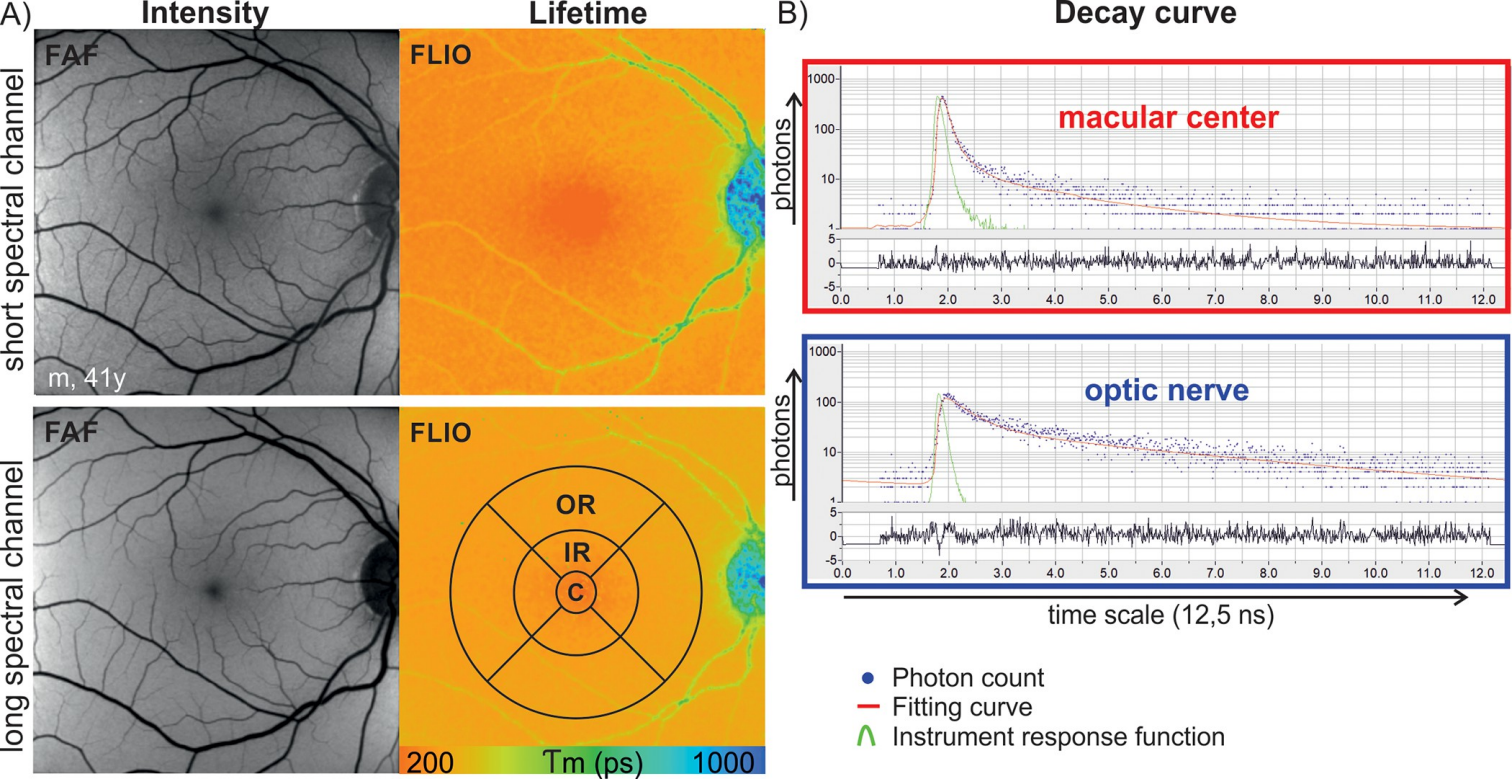
Fluorescence lifetime imaging ophthalmoscopy (FLIO) in the healthy eye. A) Fundus autofluorescence intensity (FAF) and fluorescence lifetime (FLIO) images in the short (498–560 nm) and the long (560–720 nm) spectral channel. A standard ETDRS grid was used for quantitative analysis of individual grid areas (C = Center (diameter d = 1mm), IR = inner ring (d = 3mm), OR = outer ring (d = 6mm)). B) Corresponding scatter plots with overlaid decay histogram illustrate the decay times of individual locations such as the macular center (red square), and the optic nerve head (blue square). X-axis: Time line, y-axis: Number of detected photons (logarithmic scale). Below the decay curve, the corresponding residuals are displayed, indicating how precise the raw data is approximated by the fitting curve. Blue dots: Number of photons at this specific point in time at this location (binning fact(1)or = 1); red curve: Fitting line; green line: Instrument response function.

**Fig 2:**
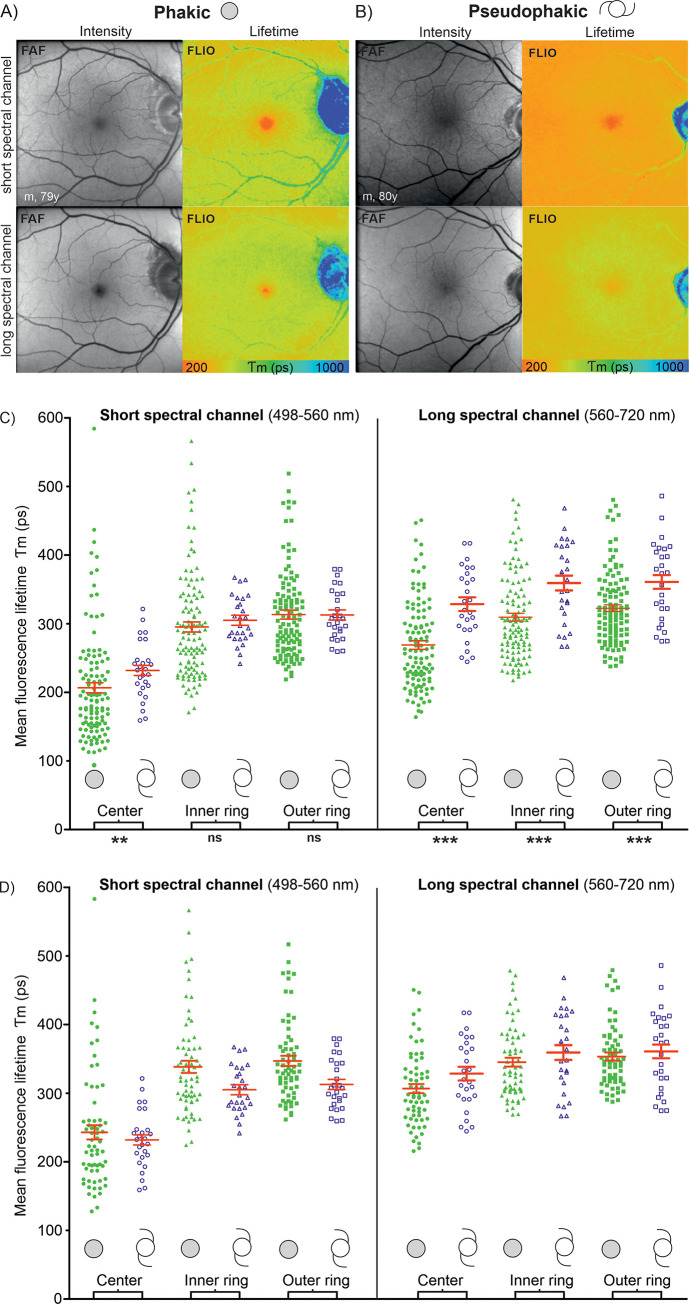
Fluorescence lifetimes in phakic and pseudophakic eyes. Comparison of retinal fluorescence lifetime in the crystalline (A) compared to the artificial intraocular (B) lens in both spectral channels. C) Diagram of mean fluorescence lifetime values (red: Mean ± SEM) in phakic (filled symbols) and pseudophakic (outlines) eyes of all age groups together. (p- values: Ns = not significant; * <0,5; * * <0,01; * * * <0,001). The age correlated distribution is shown in Fig 4. D) Subgroup analysis of subjects aged ≥ 50 years of age.

**Fig 3:**
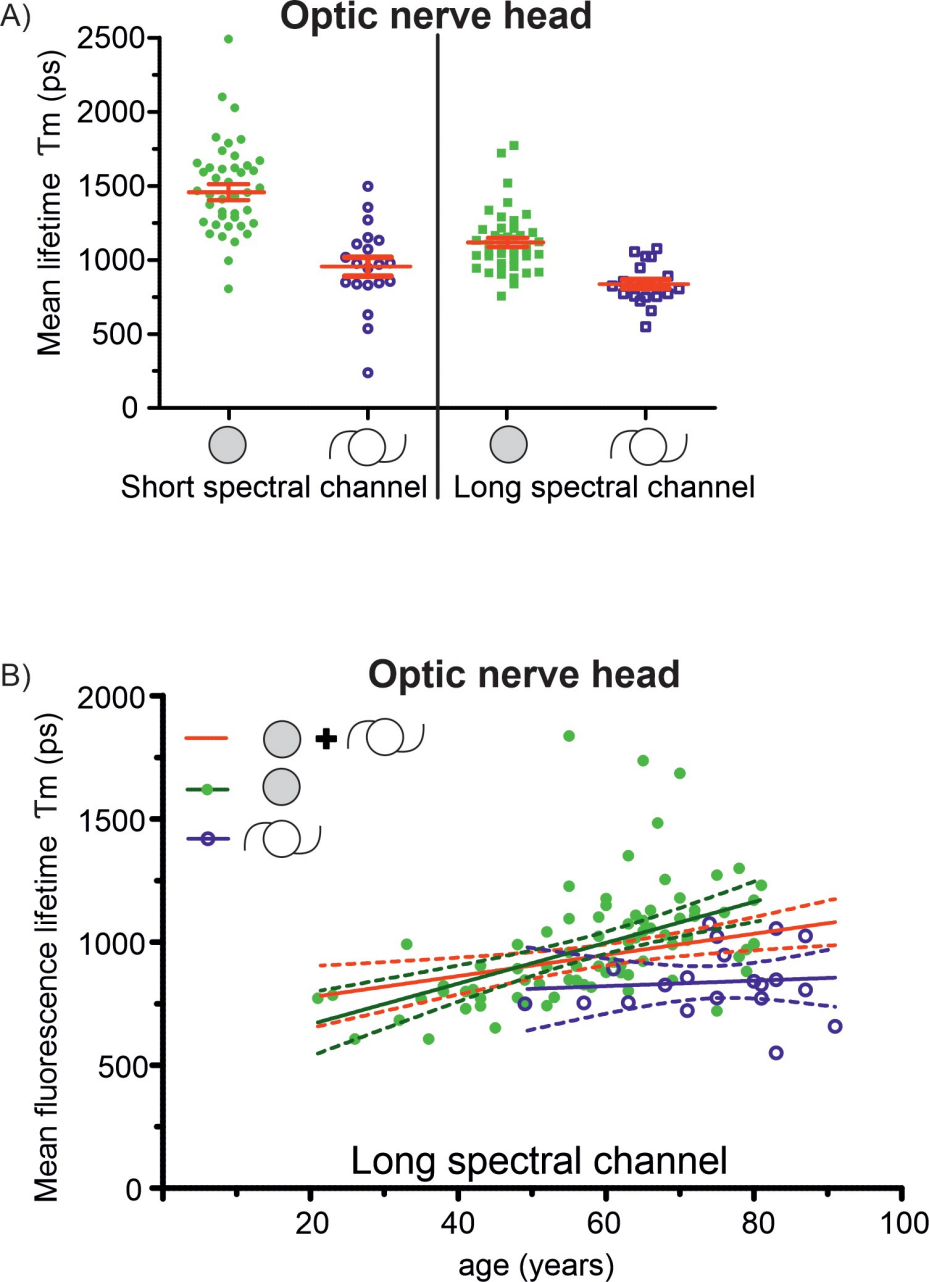
Fluorescence lifetime values of the optic nerve head. A) The mean fluorescence lifetime (*Tm*) in the optic nerve head was significantly shorter in pseudophakic compared to phakic eyes in both spectral channels (p = 0.0001 and 0.0081, resp). In the figure, only data from subjects ≥ 50 years is shown. The measured FLT value of the optic nerve in pseudophakic subjects corresponded to the FLT values of the optic nerve in healthy younger subjects. B) There was a slight increase of fluorescence lifetimes of the optic nerve head with age in phakic subjects (r^2^ = 0.26, p<0.0001). However, in pseudophakic subjects no increase of fluorescence lifetime values in the area of the optic nerve was measured (r^2^ = 0.0078, p = 0.71).

Consistent with previous studies [6,9,12] the shortest mean fluorescence lifetimes of about 210 ps in the SSC and 280 ps in the LSC were measured within the macular center (Fig 1). For detailed summary refer to Tables 2 and S1. The entire inner ring and the temporal subfield of the outer ETDRS ring featured fluorescence lifetimes of about 295 ps in the short, and 318 ps in the long spectral channel. The superior, nasal, and inferior subfield of the outer ETDRS ring showed values of about 313 ps in the short, and 480 ps in the long spectral channel (Fig 2B). The coefficient of variation was higher in the short spectral channel, towards the macular center, and in phakic eyes, whereas it was lower in the LSC, the retinal periphery, and pseudophakic eyes (Table 2). The longest FLTs were measured in the area of the optic nerve head (ONH) with values of 1220 ps in the short, and 945 ps in the long spectral channel (Fig 3). Reasons for a low coefficient of variation might be lower scattering of FLT data in longer FLT values. There were more photons collected per time in the long spectral channel compared to the short spectral channel which leads to a higher statistics for calculation of the mean FLT in the longer spectral channel and therefore also to a lower coefficient of variation. The number of subjects analyzed in the subgroup of phakic eyes is larger than in the pseudophakic group, leading to a lower coefficient of variation.

**Table 2:** Mean fluorescence lifetime values of specific regions of interest in the short (498-560nm) and the long (560-720nm) spectral channel for all subjects phakic and pseudophakic respectively.

	All					Phakic					Pseudophakic			
**Short spectral channel**	**Center**	**Inner Ring**	**Outer Ring**	**ONH**		**Center**	**Inner Ring**	**Outer Ring**	**ONH**		**Center**	**Inner Ring**	**Outer Ring**	**ONH**
Minimum	94	171	219	233		94	171	219	698		159	242	259	238
Median	197	287	302	1177		188	283	301	1246		232	297	307	974
Maximum	584	566	519	2464		584	566	519	2464		321	367	379	1496
**Mean**	**212**	**297**	**313**	**1219**		**207**	**295**	**313**	**1297**		**232**	**305**	**313**	**955**
Std. Deviation	75	71	60	376		80	77	64	352		42	34	37	283
Std. Error	6	6	5	37		8	7	6	39		8	7	7	63
Coefficient of variation	35%	24%	19%	31%		39%	26%	20%	27%		18%	11%	12%	30%
**Long spectral channel**	**Center**	**Inner Ring**	**Outer Ring**	**ONH**		**Center**	**Inner Ring**	**Outer Ring**	**ONH**		**Center**	**Inner Ring**	**Outer Ring**	**ONH**
Minimum	164	217	238	549		164	217	238	605		245	267	275	549
Median	273	307	320	903		261	297	314	959		320	364	373	826
Maximum	451	481	486	1836		451	481	481	1836		417	468	486	1075
**Mean**	**280**	**319**	**330**	**954**		**269**	**309**	**323**	**983**		**328**	**359**	**361**	**837**
Std. Deviation	65	64	57	224		63	61	54	232		51	58	58	136
Std. Error	5	5	5	22		6	6	5	26		10	11	11	30
Coefficient of variation	23%	20%	17%	23%		23%	20%	17%	24%		16%	16%	16%	16%

ONH: Optic nerve head, Std: Standard.

### Fluorescence lifetimes and age

**Fig 4:**
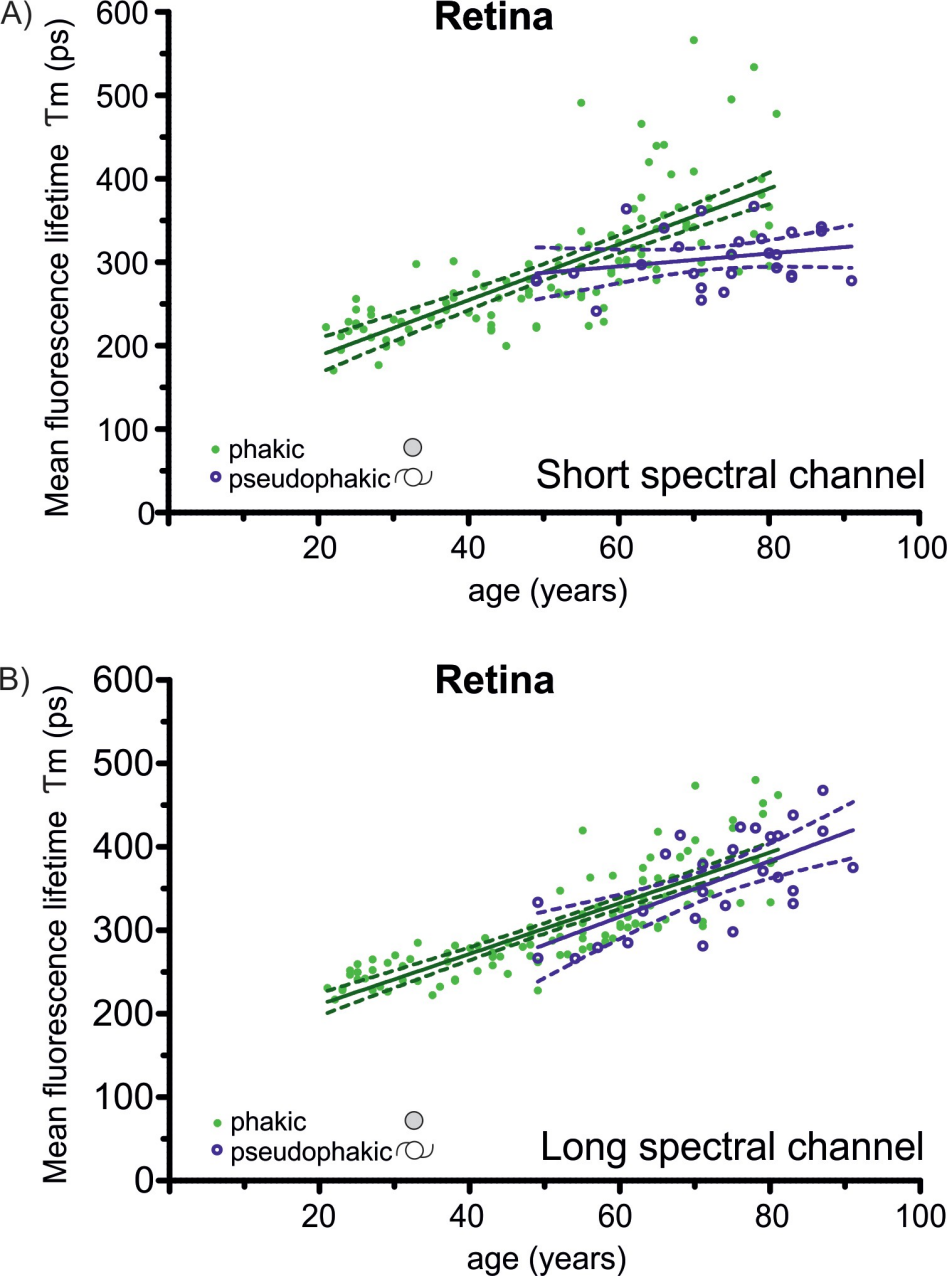
Correlation of mean fluorescence lifetimes with age. In phakic eyes, the mean fluorescence lifetime (*Tm*, ETRDS inner ring) of the retina increased with age in both spectral channel (A+B) (SSC: r^2^ = 0.54, LSC: r^2^ = 0.7; p<0.0001). In the short spectral channel (A), in phakic subjects aged around 60 years and above, a marked increase of FLT values and of the inter-individual variation were measured. In pseudophakic eyes, there was no prolongation in FLT in pseudophakic eyes in the short spectral channel (A, r^2^ = 0.066; p = 0.2). In the long spectral channel (B), in pseudophakic subjects a prolongation of FLT with age was observed, which lays in the order of the increase in phakic subjects (r^2^ = 0.44; p = 0.0002).

In phakic subjects, the mean fluorescence lifetime increased with age by about 100% in both spectral channels (205% in the SSC and 177% in the LSC; SSC: r^2^ = 0.54, LSC: r^2^ = 0.7; p<0.0001; Fig 4). This results in an increase of about 33 ps in the SSC and 28 ps in the LSC per decade. Between 20 and 40 years, the prolongation of FLT was 37% in the SSC and 27% in the LSC. In the age group between 40 and 60 years as well as in the age group between 60 and 80 years, the prolongation was around 23% in the SSC and 18% in the LSC. In the short spectral channel, there was increased scattering of individual lifetime values among subjects over 60 years, with a subgroup of measurements with long values of around 500ps.

### Fluorescence lifetimes in pseudophakic eyes

A cohort of 27 pseudophakic subjects was analyzed with an age range of 49 to 91 years (Fig 4). Within the SSC, pseudophakic subjects featured FLTs between 240 and 370 ps (mean: 305 ps) without association with age (r^2^ = 0.066; p = 0.2). In the LSC, the mean FLT increased from 285 to 420 ps with age (r^2^ = 0.44; p = 0.0002) and featured similar values as their phakic counterpart (Figs 2–4).

### Individual fluorescence lifetime parameters

**Fig 5:**
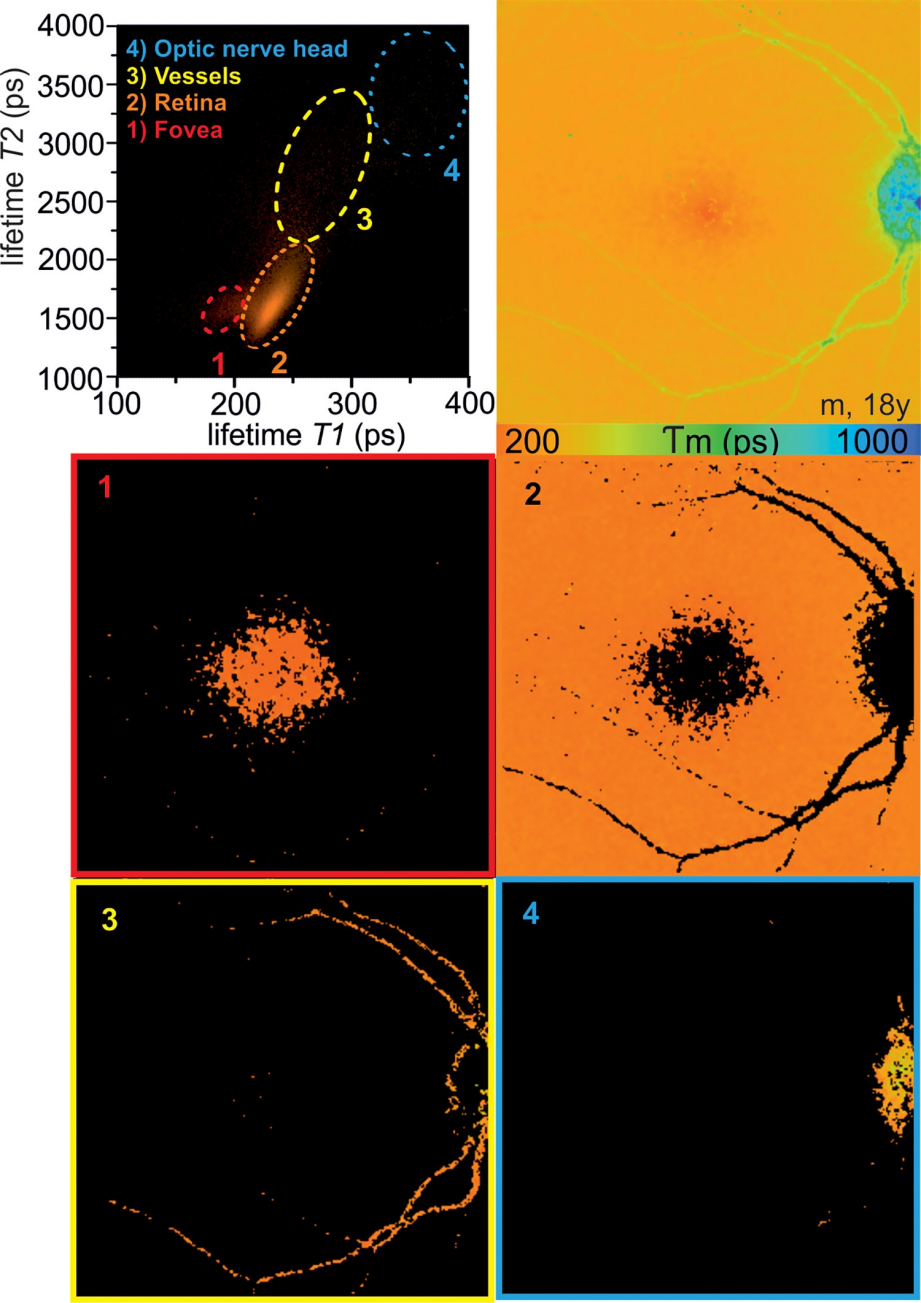
Scatter plot of fluorescence lifetime parameters. The scatter plot represents an illustrative way of depicting the interaction between the short and the long decay component within each channel. Data are shown for the long spectral channel of a phakic subject. Similar scatter plots were observed in the short spectral channel. Individual fluorescence lifetime clusters were identified and allocated to the macular center (1), the surrounding retina (2), the retinal vessels (3), and the optic nerve head (4).

A scatter plot can be used to analyze the relationship of the short and the long decay component of specific regions of interest (Fig 5). Within these plots, single lifetime clouds can be differentiated, corresponding to the macular center, the surrounding retina, the optic nerve head, and the retinal vessels.

A prolongation of fluorescence lifetimes with age was observed in both, the short (*T1*) and the long (*T2*) components of the mean decay time. In the SSC, the amplitude of the short decay component (*α1*) decreased from 97% to 94% and hence, the amplitude *α2* increased from 3% to 6%. In the LSC, there was a more pronounce decrease of *α1* from 97% to 92% and an increase in *α2* from 3% to 8%. Similar changes concerning the amplitudes are observed in both, phakic and pseudophakic subjects.

As already shown for the mean lifetime value *Tm*, there was an increase with age in the SSC and LSC in both lifetime parameters *T1* (SSC: r^2^ = 0.67, LSC: r^2^ = 0.73; p<0.0001) and *T2* (SSC + LSC: r^2^ = 0.38; p<0.0001) in phakic subjects. In pseudophakic eyes, this age dependent increase of *T1* and *T2* was only seen in the LSC but not in the SSC, which is consistent to what was seen in *Tm*.

In the area of the optic nerve head, the amplitude *α1* was about 74% in the short, and 79% in the long spectral channel. In pseudophakic eyes, the contribution of *α1* was higher (SSC: 80%; LSC: 82%), and *α2* was decreased.

### Photon distribution within the fundus

Within the macular center, a minimum of 1000 photons (mean ± SEM = 1034 ± 18 photons) was acquired in the SSC which was the channel with the slower photon counting rate in all subjects. This resulted in a nearly doubled mean photon count of 1982 ± 40 photons in the LSC. The photon ratio LSC: SSC stayed stable with progressive age. Within the optic nerve head, the LSC to SSC ratio was about one. There was no difference in the photon count ratio between phakic and pseudophakic eyes.

### Chi^2^ values

**Fig 6:**
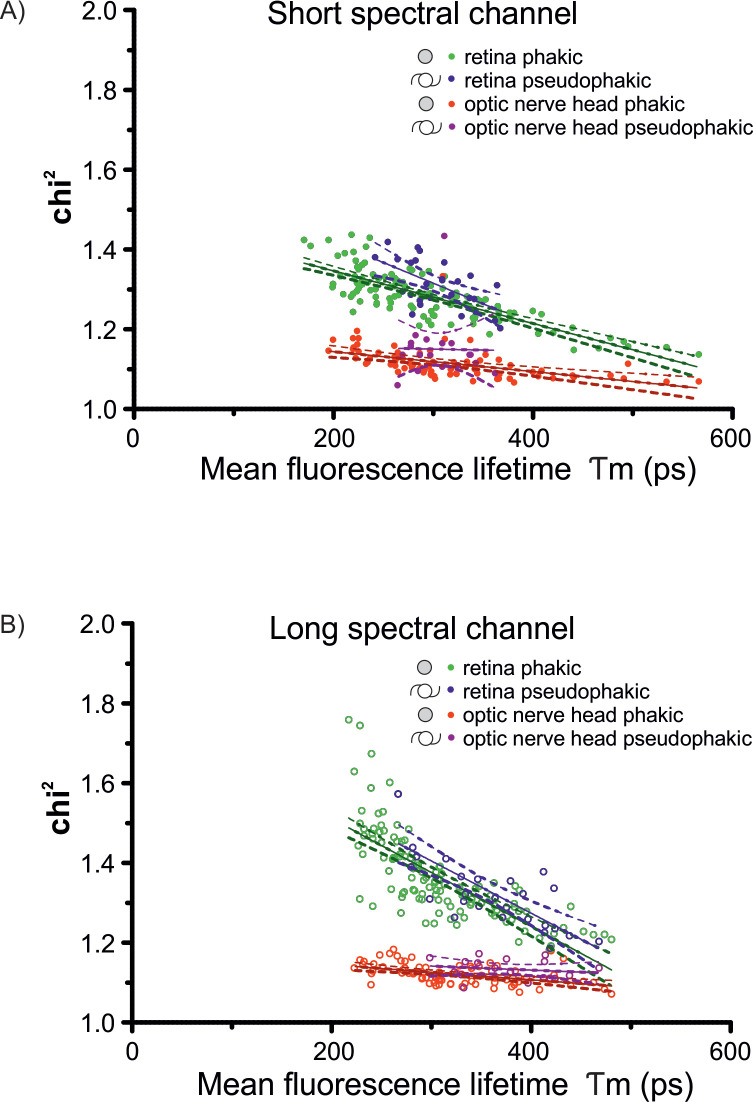
Goodness of fit (Chi^2^). In the short (A) and the long (B) spectral channel, the chi^2^ value was more precise in longer fluorescence lifetime values probably due to a larger photon count in areas of longer lifetimes. The optic nerve head featured stable chi^2^, possibly related to a constant fluorescence intensity count, independent of the FLT values.

The mean (± SEM) chi^2^ value was 1.28 (± 0.005) in the SSC and 1.36 (± 0.009) in the LSC (Fig 6). Especially in the LSC, chi^2^ was correlated with the calculated mean fluorescence lifetime, whereby the shortest FLT featured the highest chi^2^ values (SSC: r^2^ = 0.22; LSC: r^2^ = 0.52; p<0.0001). This could be explained by the lower autofluorescence intensity in the macular center, leading to a lower photon count which increases the chi^2^ value. Therefore, the chi^2^ value primarily correlates to the autofluorescence intensity and not to the autofluorescence lifetime itself. For the optic nerve head, the chi^2^ value was about 1.1 in both spectral channels and nearly remained constant over all measured mean FLTs (SSC and LSC: r^2^ = 0.01 and 0.02; p = 0.7 and 0.5). The photon count in the optic nerve head remains constant and therefore, a stable chi^2^ value can be achieved.

## Discussion

This work currently represents the largest study of fluorescence lifetime imaging ophthalmoscopy in healthy subjects and the first to systematically investigate the influence of age and the lens status on fluorescence lifetimes. In our current study including 141 healthy eyes, we can validate previous data showing the shortest FLTs in the macula with longer FLTs towards the peripheral retina, the vessels, and the optic nerve head.

By now, fluorescence lifetime imaging ophthalmoscopy (FLIO) has been studied in various retinal diseases. However, because most macular diseases represent bilaterally, intra-individual comparison of fluorescence lifetimes cannot be performed, and a healthy cohort needs to be used as control. In order to establish adequate control groups, and for longitudinal follow up of fluorescence lifetime changes, it is paramount to elucidate the influence of age and lens status on fluorescence lifetimes.

In this large cohort we found an age dependency of the mean FLT in both spectral channels of the retina, in keeping with an earlier study of our group in 31 subjects [6]. This age dependency was reproduced in other studies from independent groups [22,23]. In the present study with subjects between 21 and 91 years, an increase of about 33 ps in the SSC and 28 ps in the LSC per decade was found. This age dependent increase was observed in all ETDRS grid areas in both spectral channels, however with variation between subjects. Previous studies have shown that these age-related changes are largely due to accumulation of lipofuscin pigments within the retinal pigment epithelial cells [3,24]. This age dependency of fluorescence lifetimes in subjects over 60 years of age was largely independent of the lens status in the long spectral channel with both, phakic as well as pseudophakic eyes showing an age dependent increase of *Tm*. However, in the short spectral channel (498–560 nm) in subjects over 60 years of age, there was increased scattering of FLT data in phakic eyes. A subgroup of these phakic subjects exhibited very prolonged FLTs in the SSC. These data suggest that subclinical lens opacities lead to prolongation of FLTs within the shorter wavelength ranges between 498 and 560 nm. As pseudophakic subjects did not show significant increase in FLT with age in the SSC, no relevant age related changes of the retinal FLT is expected in this channel.

Preretinal absorption of short-wavelength light is predominantly caused by age related changes of the lens [25,26]. It has been shown that reduction and therefore scattering of light transmittance by the aging lens is predominantly affected within the short wavelengths of the visible spectrum [27]. This would explain the marked increase in FLTs in older phakic subjects within the SSC. Another factor may be lens autofluorescence which may contribute to altered FLTs in the SSC. In view of this, some authors have proposed a generalized correction and suppression of lens autofluorescence when measuring retinal FLTs [28].

As mentioned above, FLTs are not altered by age dependent lens changes in the long spectral channel. However, in the LSC, an increase of FLTs with age can be observed in phakic as well as in pseudophakic eyes. This point towards age related changes primarily in occurring in the retina. According to Greenberg et al. who investigated quantitative autofluorescence changes in healthy subjects in a wide age range [29], the main contributor to increased quantitative autofluorescence intensity is accumulation of lipofuscin. The autofluorescence intensity distribution in healthy and diseased retina has been described in detail some time ago. In a healthy retina, the macular pigment in the macula leads to a central decrease in autofluorescence intensity. Accumulation of lipofuscin and bisretinoids with increasing age have already been reported in vitro and in vivo before [3,30,31].

Detailed analysis of individual decay parameters (*T1* and *T2*, respectively *α1 and α2*) showed a shift with a decrease of the short amplitude *α1* and a corresponding increase of the long amplitude *α2*. Therefore, beside a slight general increase of T1 and T2 with age in phakic subjects, long lifetime parameters are additionally more pronounced through their larger amplitude in aging subjects. Analysis of individual lifetime parameters might help to differentiate groups of fluorophores according to their characteristics. However, by now, there can only be speculated about the origin of FLT changes, and further studies are used in this field, connecting in vitro data analysis with clinical findings.

As shown in this study, the prolongation of FLT with age in pseudophakic subjects is mainly measured in retinal FLT but not in the FLT of the optic nerve head in the LSC. Conversely, one could assume that if there is a prolongation in FLT measured on the optic nerve head in phakic subjects over time, this FLT difference might be assigned to changes in the lens.

Principally, FLTs should not be affected by the concentration of individual fluorescent compounds. However, for each pixel, collection of fluorescence lifetime data results in a decay trace whereof a decay curve is fitted, which finally results in a single lifetime value. As every single location represents a bulk response of different fluorophores, the resulting mean lifetime might be influenced and shifted towards the lifetime of a predominant fluorophore. This may explain the observed shift towards longer mean fluorescence lifetimes with age. As the emission spectrum of lipofuscin is found between 510–700 nm [7,32], particularly the FLTs in the LSC will be affected.

On the other hand, when analyzing the short emission spectrum, the crystalline lens and the cornea especially absorbs and filters out the short wavelengths light from UV light until near visible wavelengths. Therefore, the SSC between 498 and 560 nm are likely to be influenced by the lens status. By comparing photochemical properties of UV filter molecules in the human lens, the composition of different proteins has been shown to change with age [33]. Another study showed changes in the organization of cortical and nuclear lens lipid membranes as functions of age and cholesterol [34]. Additionally, metabolomic analysis points towards reduced activity and increased concentration/deposits of metabolites with age, possibly caused by dysfunction of lens epithelial cells [35].

The observation that the SSC is dependent on the lens status whereas the LSC is not, considerably influences the way of data analysis when performing FLIO studies in retinal pathologies. Namely, in both channels, the influence of age has to be considered, and control eyes have to be in the same age range. In optimal conditions–if possible–the healthy contralateral eye can be used as control. For the SSC, the influence of lens opacities has to be considered, and caution should be exercised when comparing data from phakic with pseudophakic subjects. Whether blue-blocking intraocular lenses have an influence on FLTs remains to be investigated. In patients with clinical significant lens opacities, FLTs in both spectral channels may be altered. The limitations for each wavelength detection range need to be kept in mind when investigating retinal FLTs. Generally the LSC seems to be more robust and less prone to lens artefacts. However, some FLT changes, such as macular pigment, provide better contrast in the SSC.

This study mainly addresses the characterization and quantification of possible changes originating from the crystalline lens in order to understand the measured FLT values, to include the findings in the interpretation of further FLIO images, and to be aware of possible changes of FLT which do not origin from the retina. However, lens density is a continuing challenge to fundus autofluorescence imaging in general. This holds true especially for FLIO imaging despite previous efforts to suppress artifacts of e.g. cataract on measured FLT [36].

This study has some limitations. First of all, the limitation to only two spectral channels makes it difficult to generalize the findings to a more gradual spectral range. As such the influence of lens and age may be limited to much smaller wavelength widths. Secondly, the influence of more pronounced optical absorbance, for example by cataract or corneal opacities, remains unclear and needs to be investigated. Additionally, the small sample size of the subgroup of pseudophakic subjects limits the possibility for further subgroup analysis and increases the scattering in this group. According to the expected distribution of age in pseudophakic subjects, only limited numbers are available from subjects in younger age categories. Findings gained in this study could be further explored in larger studies.

## Conclusion

Retinal autofluorescence lifetimes are influenced by age and lens status. Whereas fluorescence lifetimes within the short spectral channel are mainly affected by the lens status, fluorescence lifetimes within the long spectral channel are more prone to be influenced by age. This needs to be kept in mind when conducting studies on retinal fluorescence lifetime with FLIO.

## Supporting information

S1 Fig Age distribution in phakic and pseudophakic eyes (mean ± standard error of the mean [SEM]). (TIF)

S1 Table Mean fluorescence lifetime values (in ps) per ETDRS grid segment. Center (C), nasal (N), superior (S), temporal (T), inferior (I); (1) stands for a segment from the inner ETDRS ring, (2) stands for a segment in the outer ETDRS ring.
(DOCX)

## Abbreviations

*α1*: amplitude short lifetime component
*α2*: amplitude long lifetime component
ETDRS: Early Treatment Diabetic Retinopathy Study
FLIO: fluorescence lifetime imaging ophthalmoscopy
FLT: fluorescence lifetime
IR: infrared
LSC: long spectral channel
OCT: optical coherence tomography
RPE: retinal pigment epithelium
SD: standard deviation
SEM: standard error of the mean
SSC: short spectral channel
*Tm*: mean fluorescence lifetime
*T1*: short fluorescence lifetime component
*T2*: long fluorescence lifetime component
