# Supplementary material for: Fluorescence lifetime distribution in phakic and pseudophakic healthy eyes
Source: PLoS One. 2023 Jan 6;18(1):e0279158. doi: 10.1371/journal.pone.0279158 (PMC9821472; doi:10.1371/journal.pone.0279158)
Supplement: S1 Table — Center (C), nasal (N), superior (S), temporal (T), inferior (I); (1) stands for a segment from the inner ETDRS ring, (2) stands for a segment in the outer ETDRS ring. (DOCX) [file pone.0279158.s002.docx]

**S1 Table. Mean fluorescence lifetime values (in ps) per ETDRS grid segment.**

Center (C), nasal (N), superior (S), temporal (T), inferior (I); (1) stands for a segment from the inner ETDRS ring, (2) stands for a segment in the outer ETDRS ring.

|  | **short spectral channel** | **C** | **N1** | **N2** | **S1** | **S2** | **T1** | **T2** | **I1** | **I2** | **Inner Ring** | **Outer Ring** | **Optic Nerve Head** |
| --- | --- | --- | --- | --- | --- | --- | --- | --- | --- | --- | --- | --- | --- |
|  | **Minimum** | 94 | 167 | 217 | 184 | 232 | 164 | 206 | 169 | 213 | 171 | 219 | 233 |
|  | **Median** | 197 | 287 | 317 | 297 | 308 | 279 | 283 | 291 | 306 | 287 | 302 | 1177 |
|  | **Maximum** | 584 | 586 | 567 | 590 | 550 | 526 | 466 | 564 | 499 | 566 | 519 | 2464 |
|  | **Mean** | 212 | 296 | 327 | 309 | 320 | 287 | 291 | 297 | 316 | 297 | 313 | 1219 |
|  | **Std. Deviation** | 75 | 72 | 68 | 74 | 63 | 68 | 51 | 71 | 60 | 71 | 60 | 376 |
|  | **Std. Error** | 6 | 6 | 6 | 6 | 5 | 6 | 4 | 6 | 5 | 6 | 5 | 37 |
|  | **Coefficient of variation** | 35% | 24% | 21% | 24% | 20% | 24% | 18% | 24% | 19% | 24% | 19% | 31% |
|  |  |  |  |  |  |  |  |  |  |  |  |  |  |
|  | **long spectral channel** | **C** | **N1** | **N2** | **S1** | **S2** | **T1** | **T2** | **I1** | **I2** | **Inner Ring** | **Outer Ring** | **Optic Nerve Head** |
|  | **Minimum** | 164 | 215 | 241 | 219 | 241 | 217 | 226 | 217 | 236 | 217 | 238 | 549 |
|  | **Median** | 273 | 307 | 330 | 309 | 318 | 302 | 305 | 306 | 322 | 307 | 320 | 903 |
|  | **Maximum** | 451 | 479 | 522 | 490 | 494 | 471 | 458 | 485 | 484 | 481 | 486 | 1836 |
|  | **Mean** | 280 | 318 | 342 | 322 | 332 | 315 | 315 | 320 | 332 | 319 | 330 | 954 |
|  | **Std. Deviation** | 65 | 64 | 61 | 65 | 58 | 63 | 52 | 63 | 56 | 64 | 57 | 224 |
|  | **Std. Error** | 5 | 5 | 5 | 5 | 5 | 5 | 4 | 5 | 5 | 5 | 5 | 22 |
|  | **Coefficient of variation** | 23% | 20% | 18% | 20% | 18% | 20% | 17% | 20% | 17% | 20% | 17% | 23% |
